# Impact of serum levels of IL-18 and soluble IL-2 receptor on the clinical outcome of patients with diffuse large B-cell lymphoma treated with R-CHOP regimen

**DOI:** 10.2144/fsoa-2019-0076

**Published:** 2019-08-28

**Authors:** Hussein M Khaled, Thoraya M Abdelhamid, Fouad M Abu-Taleb, Niveen M El-Hifnawi, Ahmad B Waley

**Affiliations:** 1 Department of Medical Oncology, National Cancer Institute, Cairo University, Egypt; 2 Department of Medical Oncology, Faculty of Medicine, Zagazig University, Egypt; 3 Department of Clinical Pathology, National Cancer Institute, Cairo University, Egypt

**Keywords:** diffuse large B-cell lymphoma, IL-18, soluble IL-2 receptor

## Abstract

**Aim & methods:**

To assess the impact of pretreatment serum levels of IL-18 and soluble IL-2 receptor (sIL-2R) on the clinical outcome of patients with diffuse large B-cell lymphoma treated with an R-CHOP protocol. Total 73 patients were included.

**Results:**

Elevated serum IL-18 (using mean as cutoff) was associated with numerically lower complete remission, and 3-year disease-free survival rates; however, the difference was not statistically significant. Nevertheless, the 3-year overall survival rates were significantly more favorable for the lower serum level group. Correspondingly, the complete remission, 3-year disease-free survival and overall survival rates for patients with low pretreatment sIL-2R levels were significantly better than individuals with higher levels.

**Conclusion:**

There is a growing body of evidence supporting the utility of pretreatment serum levels of sIL-2R and IL-18 as prognostic factors in diffuse large B-cell lymphoma patients.

## Lay abstract

Diffuse large B-cell lymphoma (DLBCL) is the most common subtype of non-Hodgkin lymphomas in Egypt. Since the introduction of rituximab, the utility of International Prognostic Index in DLBCL patients treated in the rituximab era has been questioned. Instead, biologic prognostic factors including cytokines are increasingly being investigated to stratify DLBCL patients. In this prospective single arm Phase II study, there is evidence supporting the utility of pretreatment serum levels of IL-18 and sIL-2R, which can be easily measured in clinical practice, as potential prognostic factors that may add additional information regarding response to treatment and outcome in DLBCL patients and could help stratify poor risk patients for more aggressive treatment.

Diffuse large B-cell lymphomas (DLBCLs) are the most common non-Hodgkin lymphomas (NHLs) in adults [1]. In Egypt, DLBCL represents 55% of NHLs [2]. Before rituximab, the International Prognostic Index (IPI) was considered the most reliable prognostic index for NHL patients [3]. However, in the rituximab era, IPI failed to distinguish the four risk groups previously identified in chemotherapy-alone based treatment [4]. Therefore, concerns regarding the utility of IPI were raised [5].

IL-18 is a cytokine that has been shown to affect the immune defense against tumor cells [6]. It has been reported that serum IL-18 may be a useful marker for monitoring outcome in some malignancies including gastric carcinoma [7], colon carcinoma [8] and NHL [9].

Soluble IL-2 receptor (sIL-2R) is the soluble form of IL-2 receptor that plays important roles in lymphocytes activation [10]. Some lymphoid malignancies including lymphoblastic leukemia [11], Hodgkin's disease [12], B-cell chronic lymphocytic leukemia [13], adult T cell leukemia [14] and peripheral T cell lymphoma unspecified [15] displayed elevated levels of sIL-2R. Serum levels of IL-18 and sIL-2R were found to be prognostic factors for DLBCL patients [5,16–20].

This study aimed to assess the impact of serum levels of IL-18 and soluble IL-2 receptor on the clinical course and in determination of the treatment outcome in DLBCL patients treated with R-CHOP, considering disease-free survival (DFS) and overall survival as primary end points, and complete remission (CR) rate and treatment related toxicity as secondary end points.

## Patients & methods

### Patients

This study included 73 patients with CD20-positive DLBCL who presented to the Medical Oncology Department, Zagazig University Hospital, between November 2014 and November 2018. Patients were eligible when their age was 18 years or older with no prior radiotherapy or chemotherapy in the absence of any medical contraindications for receiving R-CHOP. Patients were excluded if they were pregnant, lactating or positive for HIV. Patients with 1ry central nervous system (CNS) lymphoma or Composite lymphoma were also excluded.

### Pretreatment evaluation

Pretreatment evaluation included history; physical examination; laboratory studies (blood counts, LDH and liver and kidney functions), and virology serology (HCV Ab, HBsAg, HBcAb, PCR for serologically positive patients); and bone marrow biopsy when there was unexplained cytopenia. Staging radiology comprised CT scans of chest, abdomen and pelvis; and PET-CT scan for all patients. Patients were staged according to Lugano modification of Ann Arbor staging system. Performance status was reported according to Eastern Cooperative Oncology Group performance scale, and we used standard IPI to stratify our patients into different prognostic groups.

Pretreatment serum levels of IL-18 were assessed using Human IL-18, IL-18 ELISA Kit (E0064h) from EIAAB science (Wuhan, China). For assessment of serum levels of sIL-2R, we used humans IL-2-receptor, sIL-2R ELISA Kit (BE51121) from IBL international (Hamburg, Germany).

### Treatment plan

Patients meeting eligibility criteria were scheduled to receive R-CHOP therapy (rituximab 375 mg/m^2^ day 1, cyclophosphamide 750 mg/m^2^ day 1, doxorubicin 50 mg/m^2^ day 1 vincristine 1.4 mg/m^2^ day 1, prednisone 100 mg PO daily for 5 days) repeated at 21-day intervals. Six cycles of R-CHOP-21 were planned for patients with stage I or II disease. Patients with advanced stage disease (III, IV) were scheduled to receive six to eight cycles of R-CHOP-21. Locoregional RT (36 Gy) was prescribed for patients with bulky disease (≥7.5 cm).

### Cut-off level definition

We evaluated the optimal cut-off values for serum IL-18 and sIL2R levels to predict CR using the area under the receiver operating characteristic (ROC) curve. In the current study, the ROC curve generated cut-off value for serum IL-18 was 11.3 pg/ml. Corresponding value for sIL-2R was 592.4 U/ml. We also tested the median (10.8 pg/ml) and mean serum IL-18 level (22.2 pg/ml) to determine the most appropriate cut-off value for serum IL-18.

### Statistical analysis

SPSS, version 25 (SPSS Inc., IL, USA) was used for data management and analysis. Chi-square or Fisher exact tested proportion independence. Correlation analysis was used to show strength and significance of association between quantitative variables. Kaplan–Meier method estimated overall survival (OS) and DFS and log rank test compared survival curves. Multivariate analysis was done by Cox regression model. P value was considered significant at ≤0.05 levels. Overall survival was calculated from the date of diagnosis to date of death or last follow-up. Disease-free survival was calculated as the period the patient lived without evidence of disease relapse or death.

## Ethical conduct

The authors state that they have obtained appropriate institutional review board approval from the Egyptian National Cancer Institute, Cairo University and have followed the principles outlined in the Declaration of Helsinki for all human experimental investigations. In addition, informed consent has been obtained from the participants involved.

## Results

### Baseline characteristics

Clinical characteristics of the 73 patients included are listed in Table 1. The mean ± standard deviation (SD) of the serum IL-18 level was 22.2 ± 36.3 pg/ml (range = 3.8–231.7) with a median of 10.8 pg/ml. On the other hand, the mean ± SD of the serum sIL-2R level was 732.3 ± 645.6 U/ml (range = 67.5–2362.6) with a median of 468.8 U/ml.

**Table 1.** Patients characteristics.

Variable	Frequency (n = 73)	Percentage
Gender – Males – Females	44 29	60% 40%
Median age (years) (Range)	53 (18xx68)	–
ECOG PS – PS 0 – PS 1 – PS 2 – PS 3	26 32 10 5	36% 44% 14% 6%
LDH level – Normal – Elevated	31 42	42.5% 57.5%
Number of extranodal sites – 0 – 1 – 2 or more	44 24 5	60% 33% 7%
Extranodal sites – Stomach – Bone marrow – Liver – Chest wall – Muscle – Pancreas – Paraspinal – Bone – Breast – Paratoid – Kidney – Lung – Small intestine	9 6 5 3 2 2 2 1 1 1 1 1 1	-
Stage I Stage II Stage III Stage IV	10 27 17 19	14% 37% 23% 26%
IPI risk score – Low – Low intermediate – High intermediate – High	34 21 12 6	47% 29% 16% 8%
B symptoms – Absent – Present	48 25	66% 34%
Bulky disease (≥7.5 cm) – Absent – Present	60 13	82% 18%
*HBsAg* −ve +ve *HBcAB* −ve +ve *HBV* DNA Not detectable Detectable	72 1 73 0 1 0	98% 2% 100% 0% 100% 0%
*HCVAb* −ve +ve *HCV* RNA Not detectable Detectable	43 30 20 10	59% 41% 77% 33%

ECOG: Eastern Cooperative Oncology Group; IPI: International Prognostic Index; PS: Performance status.

### Response to chemoimmunotherapy & survival

Complete response was achieved in 76% (54/72), while no CR (PR, SD and PD) was reported in 34%. After a median follow-up time of 15 months (range = 1–44 months), the 3-year OS rate was 72.9%. The median OS was not reached. The 3-year DFS rate for the 54 patients who achieved CR was 60.5%. At the end of follow-up, 20% of patients suffered relapse.

### Association of serum cytokines with clinical features

Serum IL-18 levels were significantly associated with elevated LDH (p = 0.017), but not with other poor prognostic indicators or HCV seropositivity. On the other hand, various poor prognostic indicators, such as being elderly, increased LDH, advanced disease, existence of B symptoms and unfavorable IPI were associated with high serum sIL-2R levels; but not with poor PS, multiple extranodal sites, bulky disease or HCV seropositivity (Table 2).

**Table 2.** Serum IL-18 level according to conventional prognostic factors.

Factor		Serum IL-18			Serum sIL-2R		
		Median	Range	p-value	Median	Range	p-value
All patients		10.8	3.8 – 231.7		468.8	67.5 – 2362.6	
Gender	Males	10.8	3.8 – 231.7	0.739	456.8	67.5 – 2331.6	0.955
	Females	10.8	4.8 – 110.5		468.8	94.8 – 2362.6	
Age	≤60	9.9	3.8 – 159	**0.052**	355.3	67.5 – 2362.6	**<0.001**
	>60	13.5	5.8 – 231.7		1145.7	98.7 – 2331.6	
PS	0 or 1	10.6	3.8 – 231.7	0.465	454.7	67.5 – 2362.6	0.594
	2 – 4	11.0	5.6 – 86.5		468.8	104.3 – 2096.2	
LDH	Normal	8.5	3.8 – 110.5	**0.017**	330	67.5 – 1942.2	**<0.001**
	Elevated	12.8	4.8 – 231.7		799.2	104.3 – 2362.6	
Number of extranodal sites	0 or 1	10.9	3.8 – 231.7	0.243	422.7	67.5 – 2231.6	0.078
	≥2	9.3	5.6 – 28.5		766.4	478.8 – 2362.6	
Clinical stage	I, II	9.0	3.8 – 110.5	0.066	350.0	67.5 – 1942.2	**0.001**
	III, IV	12.8	4.8 – 231.7		799.2	104.3 – 2362.6	
IPI	L	8.9	3.8 – 110.5	0.615	310.9	67.5 – 1942.2	**<0.001**
	LI	12.1	5.8 – 159		725.6	104.3 – 1990.5	
	HI	13.5	5.6 – 231.7		1819.6	541.8 – 2362.6	
	H	22.2	9.3 – 79.1		1989.2	478.8 – 2080	
B symptom	(−)	10.9	3.8 – 159	0.949	376.8	94.8 – 2231.6	**0.036**
	(+)	10.6	4.8 – 231.7		725.6	67.5 – 2362.6	
Bulky sites	(−)	9.9	3.8 – 231.7	0.231	451.8	67.5 – 2362.6	0.171
	(+)	13.8	4.8 – 79.1		598.0	171.7 – 2231.6	
HCV Ab	(−)	8.63	3.8 – 159	0.438	414.7	67.5 – 2080	0.069
	(+)	13.8	5.6 – 231.7		725.6	98.7 – 2362.6	

Values in bold highlight variables with significant p value.

HCV Ab: Hepatitis C virus antibodies; IPI: International prognostic index; LDH: LDH level; PS: Performance status.

### Univariate & multivariate analyses for cytokines & conventional prognostic variables on remission rates & survival outcomes

Utilizing ROC curve cut-off value (11.3 pg/ml), high serum levels of IL-18 were associated with numerically lower CR rate compared with lower levels (69.74 vs 79.5% [p = 0.339]). Similar results were obtained when we stratified patients according to the median (10.8 pg/ml) and the mean (22.2 pg/ml) serum IL-18 level. On the other hand, the CR rates for patients with sIL-2R levels ≤592.4 U/ml were significantly better than patients with higher serum levels (90.9 vs 50.0% [p = 0.001]). Additionally, the CR rates were significantly worse in patients with elevated LDH, advanced stage and unfavorable IPI, whereas bulky disease was marginally associated with lower CR rates. Conversely, the CR rates were not significantly associated with other clinical features, such as being elderly, poor PS, multiple extranodal sites, existence of B symptoms or HCV seropositivity (Table 3).

**Table 3.** Remission rate and survival outcome according to serum levels IL-18 and sIL-2R and other prognostic factors.

Factor		Number	CR rate		3-year DFS		3-year OS	
		Percentage	p-value	Percentage	p-value	Percentage	p-value	
Serum IL-18 (ROC)	≤11.3 pg/ml	39	79.5	0.339	49.3	0.279	62.9	0.153
	>11.3 pg/ml	34	69.7		66.2		72.1	
Serum IL-18 (mean)	≤22.2 pg/ml	60	76.7	0.465	72.1	0.127	77.8	**0.008**
	>22.2 pg/ml	13	66.7		NR		55.4	
Serum IL-18 (median)	≤10.8 pg/ml	37	78.4	0.496	40.9	0.126	54.8	0.280
	>10.8 pg/ml	36	71.4		70.1		73.7	
Serum sIL-2R	≤592.4 U/ml	44	90.9	**0.001**	75.4	**0.007**	95.4	**0.001**
	>592.4 U/ml	29	50.0		35.4		NR	
Gender	Males	44	79.5	0.264	53.4	0.131	68.1	0.854
	Females	29	67.9		93.3		86.1	
Age	≤60	52	80.4	0.100	67.4	0.581	83.9	0.124
	>60	21	61.9		55.0		49.8	
PS	0 or 1	58	78.9	0.132	65.0	0.385	72.7	0.116
	2–4	15	60		NR		71.8	
LDH	Normal	31	87.1	**0.039**	69.8	0.975	100	**0.014**
	Elevated	42	65.9		36.0		59.0	
Number of extranodal sites	0 or 1	68	77.9	^†^	60.5	^†^	74.3	**0.038**
	≥2	5	25.0		NR		NR	
Clinical stage	I, II	37	86.5	**0.021**	81.5	**0.008**	82.6	**0.012**
	III, IV	36	62.9		NR		61.4	
IPI	L	34	88.2	**0.003**	85.3	^†^	97.0	**0.001**
	LI	21	76.0		NR		55.6	
	HI	12	45.5		NR		55.6	
	H	6	0.0		NR		NR	
B symptoms	(−)	48	77.1	0.564	65.5	**0.053**	84.8	**0.010**
	(+)	25	70.8		56.9		55.4	
Bulky sites	(−)	60	79.7	**0.052**	NR	0.314	69.9	0.414
	(+)	13	53.8		83.3		68.4	

Values in bold highlight variables with significant p value.

† p-value cannot be calculated because of small numbers within some strata.

DFS: Disease-free survival; IPI: International prognostic index; LDH: LDH level; PS: Performance status; NR: Not reached; OS: Overall survival; ROC: Receiver operating characteristic.

**Figure 1.**
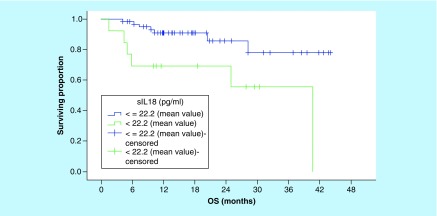
Overall survival according to the mean of serum level of IL-18 (p = 0.008). OS: Overall survival.

Serum IL-18 level was not predictive of OS using ROC curve value or the median serum IL-18 level as cut-off points (Table 3). Using the mean serum IL-18 level as a cutoff, the 3-year OS rates for patients with low and high serum levels were 77.8 and 55.4%, respectively, this difference was statistically significant (p = 0.008; Figure 1).

**Figure 2.**
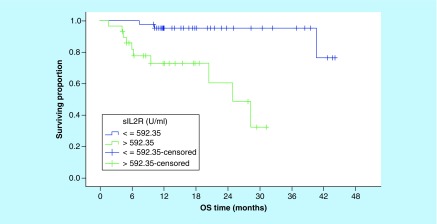
Overall survival according to serum level of sIL-2R (p = 0.001). OS: Overall survival.

**Figure 3.**
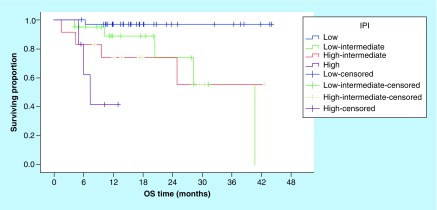
Overall survival according to International Prognostic Index (p = 0.001). IPI: International prognostic index; OS: Overall survival.

Correspondingly, the 3-year OS rates for patients with sIL-2R levels ≤592.4 U/ml were significantly better compared with patients with higher serum levels (95.4% vs NR, [p = 0.001]; Figure 2). In addition, the OS rates were significantly worse in patients with increased LDH, multiple extranodal involvement, advanced stage, presence of B symptoms (Table 3) and unfavorable IPI (Figure 3).

**Figure 4.**
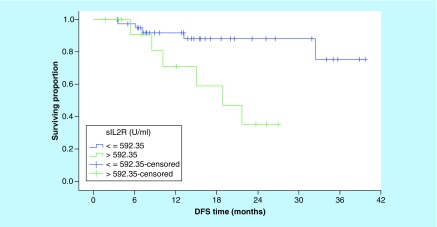
Disease-free survival according to serum level of sIL-2R (p = 0.007). DFS: Disease-free survival.

**Figure 5.**
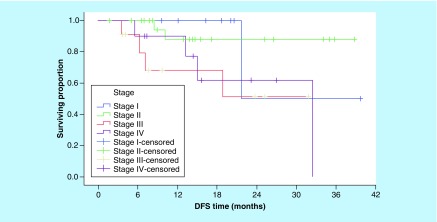
Disease-free survival according to clinical stage (p = 0.008). DFS: Disease-free survival.

With respect of DFS, serum level of IL-18 was not predictive of 3-year DFS outcome when we used either ROC curve value, the median or the mean serum IL-18 level as cut-off points. Conversely, the patients with low serum sIL-2R had a significantly better 3-year DFS rates than patients with higher serum sIL-2R (75.4 vs 35.4%; p = 0.007; Figure 4). On the other hand, none of the conventional prognostic factors was predictive of DFS (Table 3), except for clinical stage (p = 0.008; Figure 5).

Multivariate analyses employing strong conventional prognostic factors and serum cytokine levels demonstrated that serum sIL-2R was independent prognostic factor for OS, and that clinical stage and sIL-2R were marginal independent prognostic factors for DFS (Table 4).

**Table 4.** Multivariate analyses on overall survival and disease-free survival.

Factor	HR	95% CI	p-value
Overall survival			
sIL-2R (>592.4 U/ml)	11.41	2.5 – 52.4	**0.022**
Disease-free survival			
sIL-2R (>592.4 U/ml)	4.1	1.0 – 16.5	**0.057**
Clinical stage (advanced stage)	3.5	0.9 – 12.8	**0.047**

Values in bold highlight variables with significant p-value.

HR: Hazard ratio.

## Discussion

Many investigators have attempted to identify prognostic factors to discriminate NHL patients who are likely to have favorable or worse outcomes [21].

Since the introduction of rituximab, the utility of IPI has been questioned [5]. To more strictly select appropriate therapeutic options, poorer prognostic groups need to be further discriminated.

Cytokines are small proteins that play an important role in immune regulation, and might reflect tumor growth. Currently, it is well known that an altered cytokine milieu exists in DLBCL [22].

In this study, we investigated pretreatment serum IL-18 and sIL-2R levels in DLBCL patients treated with R-CHOP as prognostic factors with respect to OS, DFS, CR rates. The thresholds used to define the prognostic groups constitute a source of debate in any study on serum cytokines. We evaluated the optimal cut-off values for serum IL-18 and sIL2R levels to predict CR using the ROC curve. With the use of these cut-off values, we divided the patients into low and high groups.

In the current study, the ROC curve generated cut-off value of serum IL-18 was 11.3 pg/ml, and this value was much lower than that reported by Goto and collegues (590 pg/ml) [18]. Correspondingly, cut-off value of sIL-2R in our patients was 592.4 U/ml, and this value was also lower than cutoff described by precedent investigators [4,20,23]. These observed variations might be explained by distinctive nature of the disease in Egyptian patients including variable etiologic, genetic and biological factors.

We conducted univariate analysis of the effect of serum IL-18 upon disease outcome end points.

Utilizing ROC curve cut-off value, elevation of serum IL-18 was not predictive of CR (p = 0.339), 3-year DFS (p = 0.279) or 3-year OS (p = 0.153) rates. This might be referred to the impact of other predictors of outcome in DLBCL that might have been overlooked in our study (immunoblastic histology; molecular markers such as BCL2, c-myc, stromal signatures, gene expression profile subtypes) [24]. Additional potential confounders include the IL‐18 binding protein (IL‐18BP) which is known affect the concentrations of active free form of IL‐18 [25,26].

The aforementioned findings are in line with the results of a Turkish group who evaluated changes in pre- and post-treatment levels of serum IL-18 in 46 aggressive NHL patients (87% of cases were DLBCL). Serum levels of serum IL-18 remained unchanged after chemotherapy. Consequently, their conclusion was that serum IL-18 measurements have no prognostic value in aggressive NHLs including DLBCL [27].

Moreover, we probed utility of mean serum IL-18 level as a predictive cut-off value. Elevation of serum IL-18 was not predictive of remission (p = 0.465), or 3-year DFS rates (p = 0.127). Nevertheless, the 3-year OS rates for patients with high and low serum IL-18 levels were significantly different (p = 0.008). Earlier, Goto and fellows reported that serum IL-18 levels were predictive of CR, 4-year PFS, and OS rates in patients treated with R-CHOP (p = 0.0048, p < 0.0001, p < 0.0001, respectively) [18]. Given these finding, serum IL-18 level seems to have a probable propensity to affect OS in DLBCL patients treated in rituximab era. However, a greater number of cases need to be followed-up for longer periods of time to reach a clearer understanding of the prognostic value of serum IL-18 level in DLBCLs in rituximab era.

On the other hand, the CR, 3-year DFS and OS rates for our patients with low pretreatment sIL-2R levels were significantly better than individuals with higher levels (p = 0.001, p = 0.007, p = 0.001, respectively). In multivariate analysis, high sIL-2R independently correlated with shorter DFS and OS (HR: 4.1, 95% CI: 1.0–16.5, p < 0.057; and HR: 11.4, 95% CI: 2.5–52.4, p < 0.022, respectively). These obtained results were like those reported by Goto and coworkers who found that high sIL-2R patients had a poorer outcome in terms of CR, PFS and OS for patients with DLBCL treated with R-CHOP (p = 0.0003, p < 0.0001, p < 0.0001, respectively) [5]. Additional subsequent studies identified the prognostic value of sIL-2R in DLBCLs patients from different age and ethnic groups treated with R-CHOP. A report from Tomita and colleagues identified the prognostic value of sIL-2R levels either individually or as a part of SIL index (Stage, sIL-2R, LDH) in predicting poor PFS, and OS outcomes in 572 DLBCL patients treated with R-CHOP [28]. Subsequently, a Spanish group evaluated the prognostic value of different cytokines including sIL-2R in 197 DLBCL patients treated with Rituximab based immunochemotherapy. Elevated levels of serum sIL-2R were significantly associated with lower CR rates and shorter PFS and OS [19]. In a like manner, prognostic impact of pretreatment serum sIL-2R level was identified in elderly patients with DLBCL patients treated with R-CHOP in a Japanese study. This study showed that measurement of the sIL-2R level at diagnosis is clinically beneficial for identifying elderly patients with DLBCL who have a poor prognosis in terms of PFS and OS [20]. Collectively, there is growing body of evidence supporting the proposal that pretreatment serum levels of sIL-2R is a potentially robust prognostic factor that can help stratifying poor risk DLBCL for more aggressive treatment.

The present study has several limitations. First, this was a study from a single institute. Second, the small sample size, and heterogeneity of patients’ characteristics in our study might have limited our ability to identify statistically significant differences in terms of OS, DFS and CR rates with respect to serum IL-18, and other conventional prognostic factors. Third, serum IL-18 and sIL-2R are not specific markers for DLBCL and its level are increased in several inflammatory conditions including viral infection and autoimmune disorders. Further, we did not consider many relevant prognostic factors, such as BCL2, MYC, Ki67 and GEP subtypes in our analyses.

## Conclusion

Being potentially effective and applicable, pretreatment serum level of sIL-2R in DLBCL patients deserves to be considered as another important tool for identifying those with poor prognosis, whereas serum IL-18 level should be studied in larger prospective studies. Additionally, the most reliable prognostic factor or the best combination of some prognostic factors for DLBCL should be further clarified in order to assist in selecting the most appropriate immunotherapy-based treatment.

## Future perspective

Our present findings have provided evidence for the utility of pretreatment serum levels of sIL-2R as potential prognostic factors in DLBCL patients. However, the most reliable prognostic factor or the best combination of some prognostic factors for DLBCL should be further clarified in order to assist in selecting the most appropriate immunotherapy-based treatment.

**Summary points**

- Biologic prognostic factors including cytokines are increasingly investigated to stratify diffuse large B-cell lymphoma (DLBCL) patients.
- This study aimed to assess the impact of pretreatment serum levels of IL-18 and sIL-2R on the clinical outcome of patients with DLBCL treated with R-CHOP protocol.
- Being potentially effective and applicable, pretreatment serum level of sIL-2R in DLBCL patients deserves to be considered as another important tool for identifying those with poor prognosis, whereas serum IL-18 level should be studied in larger prospective studies.
